# Human Tamm-Horsfall protein, a renal specific protein, serves as a cofactor in complement 3b degradation

**DOI:** 10.1371/journal.pone.0181857

**Published:** 2017-07-24

**Authors:** Diana C. J. Rhodes

**Affiliations:** Department of Anatomy, Pacific Northwest University of Health Sciences, Yakima, Washington, United States of America; University of Manchester, UNITED KINGDOM

## Abstract

Tamm-Horsfall protein (THP) is an abundant urinary protein of renal origin. We hypothesize that THP can act as an inhibitor of complement since THP binds complement 1q (C1q) of the classical complement pathway, inhibits activation of this pathway, and is important in decreasing renal ischemia-reperfusion injury (a complement-mediated condition). In this study, we began to investigate whether THP interacted with the alternate complement pathway via complement factor H (CFH). THP was shown to bind CFH using ligand blots and in an ELISA (K_D_ of 1 × 10^−6^ M). Next, the ability of THP to alter CFH’s normal action as it functioned as a cofactor in complement factor I (CFI)–mediated complement 3b (C3b) degradation was investigated. Unexpectedly, control experiments in these *in vitro* assays suggested that THP, without added CFH, could act as a cofactor in CFI-mediated C3b degradation. This cofactor activity was present equally in THP isolated from 10 different individuals. While an ELISA demonstrated small amounts of CFH contaminating THP samples, these CFH amounts were insufficient to explain the degree of cofactor activity present in THP. An ELISA demonstrated that THP directly bound C3b (K_D_ ~ 5 × 10^−8^ m), a prerequisite for a protein acting as a C3b degradation cofactor. The cofactor activity of THP likely resides in the protein portion of THP since partially deglycosylated THP still retained cofactor activity. In conclusion, THP appears to participate directly in complement inactivation by its ability to act as a cofactor for C3b degradation, thus adding support to the hypothesis that THP might act as an endogenous urinary tract inhibitor of complement.

## Introduction

Tamm-Horsfall protein (THP), or uromodulin, is a renal specific glycoprotein produced by the thick ascending limb of Henle cells,[1] and is the most abundant protein in normal human urine.[2] Approximately 25% of the molecular mass of THP is formed by carbohydrates, largely sialylated N-glycans.[3,4] THP is highly acidic, with a pI of approximately 3.5.[5]

THP appears to be multifunctional. It may protect the urinary tract from bacterial colonization since THP binds to type 1 fimbriae of *E*. *coli* and inhibits adherence of these *E*. *coli* to uroepithelial cell receptors.[6] Mice deficient in THP are more prone to experimentally induced *E*. *coli* cystitis than are wild-type mice.[7] THP may play a role in renal handling of salt. Individuals with higher urinary THP levels excrete less sodium and are more likely to have hypertension.[8] Additionally, THP may have immunomodulatory activities because of its interaction with various cytokines[9,10,11] and immune cells.[12,13,14,15,16] Immunoglobulin G and light chains also bind THP.[17,18]

We hypothesize that one function of THP is to serve as an endogenous complement inhibitor. THP binds strongly to complement 1q (C1q), a key initiating protein in the classical complement pathway, and appears to prevent activation of this pathway.[19,20,21] Several *in vivo* studies suggest a linkage between THP and renal complement inhibition. Complement activation occurs during ischemia-reperfusion injury (IRI)[22,23] and organ transplantation.[24,25] THP knockout mice were less able to protect their kidneys from tubular damage when subjected to renal IRI than were wild-type mice[26] and during the recovery phase of IRI, THP was redirected to the tubulointerstitium.[27] In liver transplant patients, patients with the lowest pre-surgical urinary THP concentrations were the ones who developed renal insufficiency post-surgically.[28] In renal transplant patients, THP urinary concentrations were significantly lower in patients that developed acute renal rejections compared to individuals with stable kidney grafts.[29]

The present study initially was aimed at exploring whether THP interacts with the alternate complement pathway via complement factor H (CFH). The main function of CFH is to protect host tissues from complement-mediated damage by disrupting the cascade of events associated with the deposition of complement 3b (C3b).[30] CFH accomplishes this complement down regulation by, among other actions, acting as a cofactor for complement factor I (CFI) in the proteolysis of C3b.[30] There are several short consensus repeats on CFH which bind to polyanions such as heparin and sialic acid complexes, including the C-terminal region of CFH which binds C3b.[31] It was hypothesized that THP, with its sialic acid residues, would bind to CFH and perhaps alter CFH’s ability to act as a cofactor in C3b degradation.

We first used ligand binding and enzyme-linked immunosorbent assays (ELISA) to demonstrate that THP bound CFH. Experiments then were conducted to determine if THP binding to CFH altered the ability of CFH to serve as a cofactor for CFI in C3b degradation. Unexpectedly, it appeared that THP, without CFH, could serve as a cofactor of CFI-mediated C3b degradation. Multiple experimental protocols were used to confirm that THP did directly act as a cofactor in CFI-mediated C3b degradation and that the protein portion of THP most likely was responsible for this activity.

## Material and methods

### Ethics statement

Written informed consent was obtained from participants who then provided urine samples for purification of the THP used in this study. This study process was approved by the Institutional Review Board of the Kirksville College of Osteopathic Medicine where these studies were initiated.

### THP purification

Human THP was purified from clean-catch, 12–24 h normal human urine samples from a total of 10 adults (5 male and 5 female subjects) by multiple NaCl precipitations as described previously.[19]

### CFH/THP ligand blot

To begin to explore the possibility that THP interacts with CFH, a ligand blot was performed. CFH (1.5 μg/lane) (Calbiochem, San Diego, CA, USA), both non-reduced and reduced (with 3% ß-mercaptoethanol (Sigma Chemical Co., St. Louis, MO, USA)) was electrophoresed on a 7.5% SDS-PAGE gel and then was transferred electrophoretically (70V, 2 h) to nitrocellulose (NC) (Immobilon, Millipore, Bedford, MA, USA). One NC panel was stained for total protein with amido black stain (Sigma Chemical Co., St. Louis, MO, USA). The remaining two NC strips were blocked with 10 mg/ml BSA in 10 mm NaPO_4_/10 mm NaCl (pH 7.55). One of these NC strips was incubated overnight with 40 μg/ml THP in 10 mg/ml BSA/ 10 mm NaPO_4_/ 10 mm NaCl/ 0.05% Tween 20 (pH 7.55). After rinsing with the incubation buffer, both blocked NC strips were incubated with sheep anti-human THP (1:500, polyclonal, The Binding Site, San Diego, CA, USA) for 3 h and with donkey anti-sheep IgG-HRP (1:1000, polyclonal, Sigma Chemical Co., St. Louis, MO, USA) for 1 h. The bound enzyme-labeled antibody was detected using 0.5% 4-chloro-1-naphthol/ 0.01% H_2_O_2_ (Sigma Chemical Co., St. Louis, MO, USA) in TBS. All incubations were performed at room temperature.

### THP ELISA’s

The ability of THP to bind CFH was explored further using an ELISA format. Microtiter, 96-well plates (Falcon Pro-Bind, Becton Dickinson, Lincoln Park, NJ, USA) were coated with 25 μg/ml human THP in 0.05 mm sodium carbonate (pH 9.6) overnight at 4°C. Human CFH (10 μg/ml to 250 μg/ml), diluted in 1% BSA/20 mm Tris/20 mm NaCl/0.05% Tween 20 (pH 7.5) (BSA/Tris buffer) was incubated in duplicate THP-coated wells (overnight, 4°C). Bound CFH was detected by sequential incubation with goat anti-human CFH (1:1000, polyclonal, Calbiochem, San Diego, CA, USA) in BSA/Tris buffer (overnight, 4°C), and Protein A/G—alkaline phosphatase (1:250, Pierce, Rockford, IL, USA) in BSA/Tris buffer (1 h at 37°C), and then with 4 mm p-nitrophenol phosphate (Sigma Chemical Co., St. Louis, MO, USA)/1 mm MgCl_2_/0.05 m sodium carbonate buffer (pH 9.6). Once the OD_405_ of the darkest wells approached 2.0, the reaction was stopped with 4 N NaOH and the absorbance of wells at 405 nm was measured. Negative control wells, those without CFH, were included on all plates and the value for non-specific binding of antibodies (~7% of maximal binding) was subtracted from the experimental wells. Additional wells were coated with 0.5, 1 and 2 μg/ml CFH to allow conversion of the OD_405_ measurements to concentrations of CFH bound, assuming a molecular weight of 150 kD for CFH.

An inhibition ELISA was used to determine if soluble THP could compete with immobilized THP for binding to CFH. The basic ELISA format described above was modified by addition of soluble THP to the CFH incubation step. A single concentration of CFH (35 μg/ml) was incubated at 4°C for 1 h with THP concentrations ranging from 0 to 200 μg/ml. These combined CFH/soluble THP samples were incubated in duplicate THP-coated wells overnight at 4°C.

For THP/C3b ELISA’s, the initial coating of wells with THP and the various incubation times and temperatures were identical to the CFH ELISA, as was the diluent used except that 1% BSA was omitted since C3b bound strongly to BSA-coated wells. Thus, no protein was added to block wells, but rather the 0.05% Tween 20 in the incubation buffer was used to decrease nonspecific binding. Human C3b (1 μg/ml to 250 μg/ml) (Complement Technology, Tyler, TX, USA) was incubated in duplicate THP-coated wells. Bound C3b was detected by sequential incubation with goat anti-human C3b (1:3000, polyclonal, Complement Technology, Tyler, TX, USA), and rabbit anti-goat IgG—alkaline phosphatase (1:2000, polyclonal, Southern Biotech, Birmingham, AL, USA) and then developed using a p-nitrophenol phosphate solution as above. A complete series of C3b concentrations were added to control wells that had not been coated with THP to monitor for nonspecific binding Additional wells were coated with 0.5, 0.1 and 0.5 μg/ml C3b to allow conversion of the OD_405_ measurements to concentrations of C3b bound, assuming a molecular weight of 176 kD for C3b.

### Assays for cofactor activity in CFI-mediated C3b degradation

THP’s ability to modify CFH’s function as a cofactor for CFI-mediated proteolysis of C3b, utilized SDS-PAGE gels to monitor the cleavage of the α-chain of C3b into two fragments.[32]

In all cofactor assays, reactions were carried out in a 10 μl volume and were stopped by addition of 10 μl 2x sample buffer with either 6% ß-mercaptoethanol or 200 mM dithiothreitol and boiling. Half of this final mixture was run on to 8% or 12% SDS-PAGE gels that then were silver-stained. Reaction mixtures contained different combinations and amounts of complement proteins (Calbiochem, San Diego, CA, USA or Complement Technology, Tyler, Texas, USA) including C3b, CFI, and CFH (after the initial experiments), and various amounts of human THP (in 20 mm Tris/ 20 mm NaCl (pH 7.5)) and were incubated at 37°C for 30 min to 4 h. Ovalbumin (Sigma, St. Louis, MO, USA) was used at 5 μg in 10 μl for one set of cofactor assays. Deglycosylated THP (dTHP), used in one set of C3b cofactor assays, was produced using a series of glycosidases on native THP where dTHP was separated from free carbohydrates as described previously.[21] It was estimated, by the change in mobility on SDS-PAGE, that about 88% of the carbohydrate was removed from dTHP and, by lectin blotting, that all of the sialic acid was removed.[21]

### ELSIA used to evaluate THP, CFI, and C3b for CFH contamination

An AssayMax Human Complement Factor H ELISA Kit (AssayPro, St. Charles, MO, USA) was utilized to test for the presence of CFH in all 10 THP samples (each adjusted to 1 mg/ml THP), as well as C3b and CFI samples (each at approximately 60 μg/ml protein concentration). Kit instructions were followed and standard and unknown samples were analyzed in duplicate wells, with the absorbance at 550 nm being subtracted from the 450 nm absorbance to obtain corrected absorbance values.

### Data analysis

The THP ELISA data were fitted by non-linear least-squares analysis using GraFit (Erithacus Software, Staines, UK) to the equation: B = (F × Cap)/(K_D_ + F); where B is the concentration of bound CFH or C3b, F is the concentration of free CFH or C3b, Cap is the concentration of binding sites, and K_D_ is the equilibrium dissociation constant.

For the CFH ELISA, a standard curve for the interval from 0 to 5 ng/ml CFH was obtained from a least-squares 2^nd^-order polynomial regression (Sigma Plot 11, Systat Software, San Jose, CA, USA) of absorbance vs. concentration of CFH for duplicate standard samples. The concentrations of CFH in the unknown samples were predicted from the quadratic equation using the coefficients obtained from this regression.

Digital photographs of gels from some of the C3b cofactor assays were analyzed using NIH ImageJ[33] to compare the intensity of the C3b fragments to other non-fragmented proteins in the same lane, to estimate differences in cofactor activity between samples. Comparison of cofactor activity in male versus female THP utilized these ImageJ band intensity ratios and a Students’ t-test (Sigma Plot 11) with the significance set at P <0.05. A correlation between the amounts of CFH in THP samples, as measured by the CFH ELISA, with the C3b band intensity ratio for each THP sample was evaluated using the Pearson correlation coefficient calculator in Sigma Plot 11.

## Results

### THP binding to CFH

**Fig 1:**
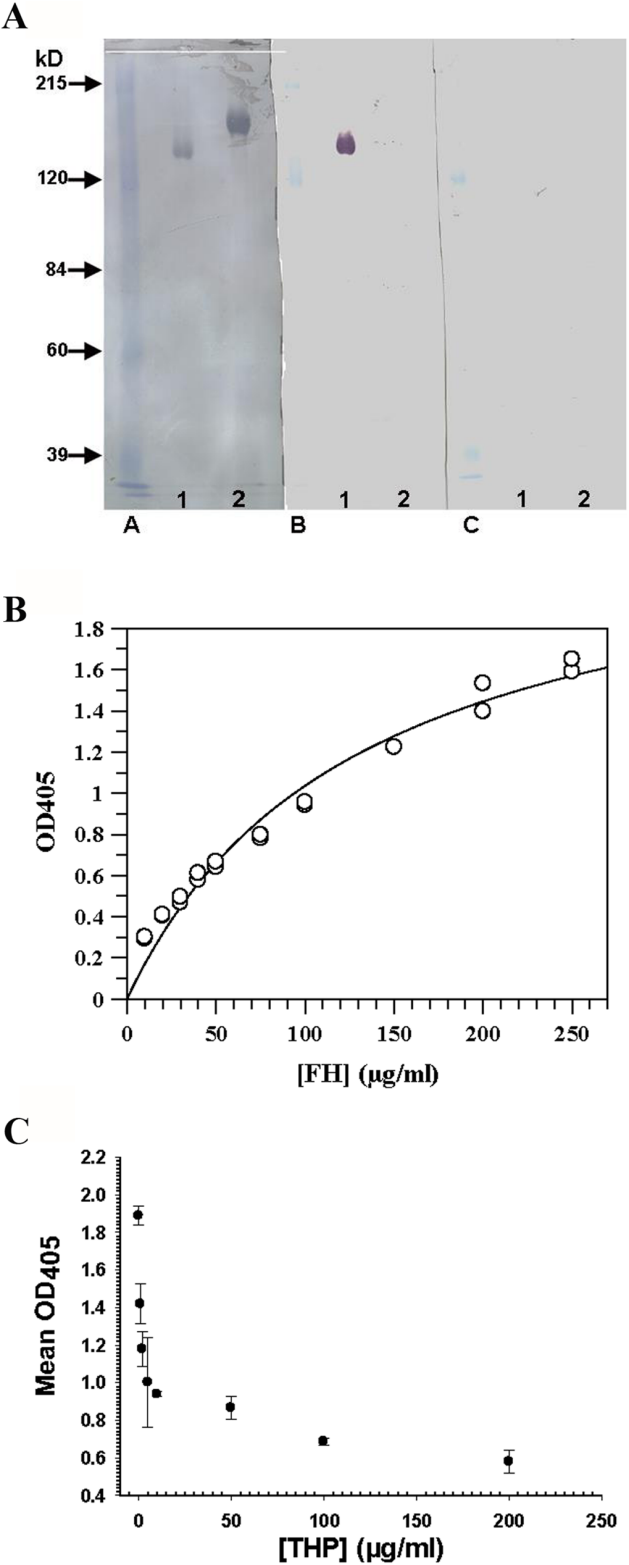
THP binding CFH. (A) THP/CFH ligand blot. Native (Lane 1) and reduced (Lane 2) CFH samples (1.5 μg/lane) were separated on a 7.5% SDS-PAGE gel and then were transferred electrophoretically to NC and reacted as follows: Panel A: stained with amido black. Panel B: incubated with THP, sheep anti-human THP, and donkey anti-sheep IgG-HRP. Panel C (negative control panel): same as panel B except without the THP incubation step. (B) THP/CFH ELISA. Results from an ELISA using ten different concentrations of CFH to bind to immobilized THP. Predicted K_D_ for this particular ELISA was 1.2 × 10^−6^ M. (C) Inhibition ELISA. Soluble THP inhibited 35 μg/ml CFH binding to immobilized THP in a dose-dependent manner.

Ligand blots demonstrated that THP bound to native, non-reduced CFH (Fig 1A, Panel B, Lane 1), but not to CFH whose disulfide bonds had been reduced (Fig 1A, Panel B, Lane 2). THP bound CFH in an ELISA (Fig 1B) with a K_D_ of 1 × 10^−6^ M. In an inhibition ELISA, soluble THP effectively competed with immobilized THP for binding to CFH (Fig 1C).

### Initial C3b cofactor assays

**Fig 2:**
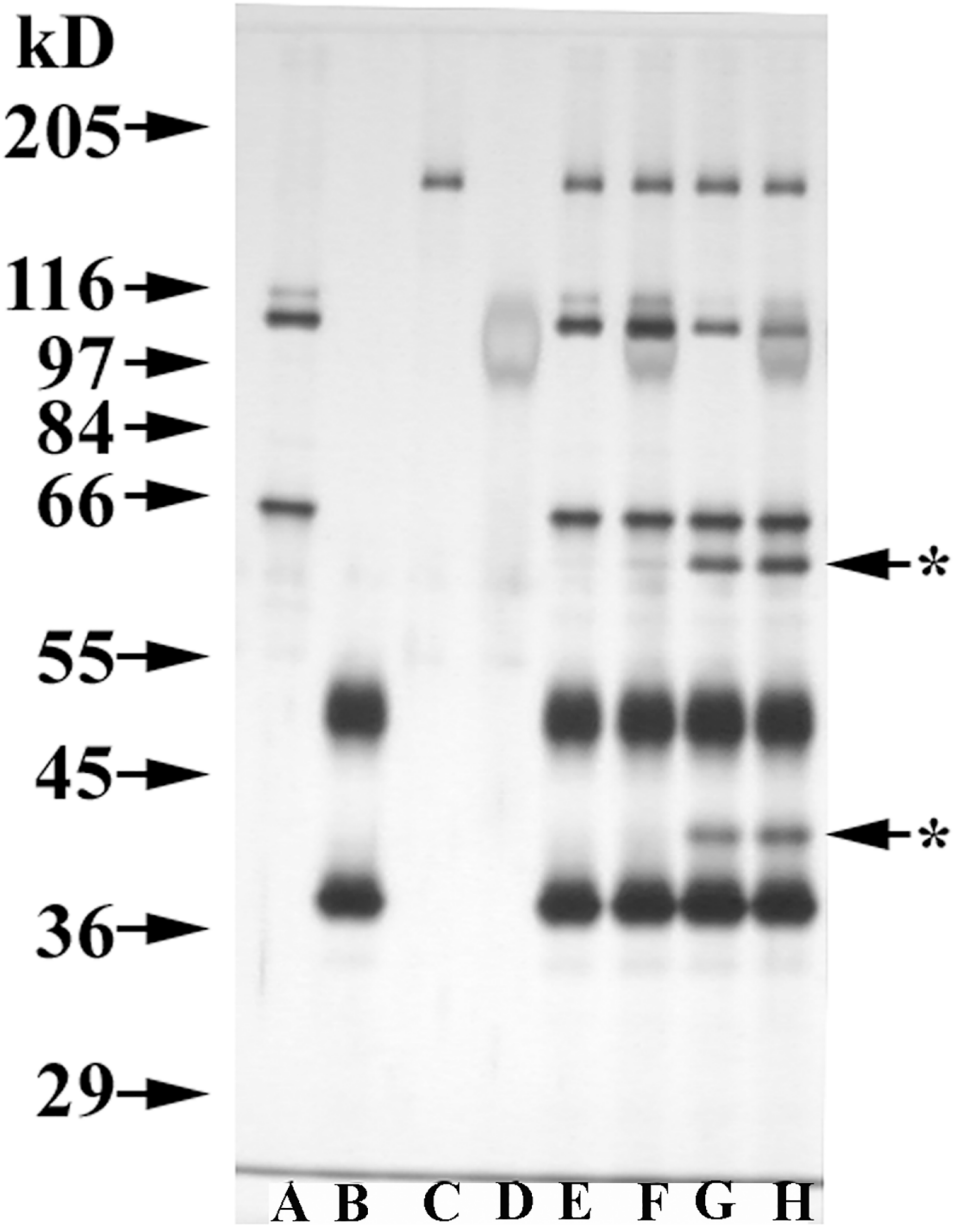
Preliminary C3b cofactor assay. All samples were reduced with 3% ß-mercaptoethanol in this 8% SDS-PAGE, silver-stained gel. Lane A: 125 ng C3b (containing its α-chain (~101 kD) and ß-chain (~75 kD)). Lane B: 500 ng CFI (with its heavy chain (~50 kD) and light chain (~38 kD)). Lane C: 50 ng CFH (~150 kD). Lane D: 500 ng THP (~100 kD). Lane E: C3b, CFI, and CFH at 37°C for 0 min. Lane F: C3b, CFI, CFH, and THP at 37°C for 0 min. Lane G: same as Lane E except incubated at 37°C for 30 min. Lane H: same as Lane F except incubated at 37°C for 30 min. Starred arrows indicate the degradation products of C3b (C3bα_62_ and C3bα_41_) present in the two samples incubated at 37°C for 30 min.

Studies were initiated to evaluate the ability of THP to modify the capacity of CFH to serve as a cofactor for CFI- mediated cleavage of C3b. Fig 2 documents the gel mobilities of the components of this assay, C3b, CFI, CFH, and THP, in individual lanes, as well as the two degradation fragments of C3b, C3bα_62_ and C3bα_41_, in samples incubated with C3b, CFI, and CFH at 37°C for 30 min either without THP (Lane G) or with THP (Lane H). C3b degradation bands were absent in control samples kept at 4°C (Lanes E and F).

**Fig 3:**
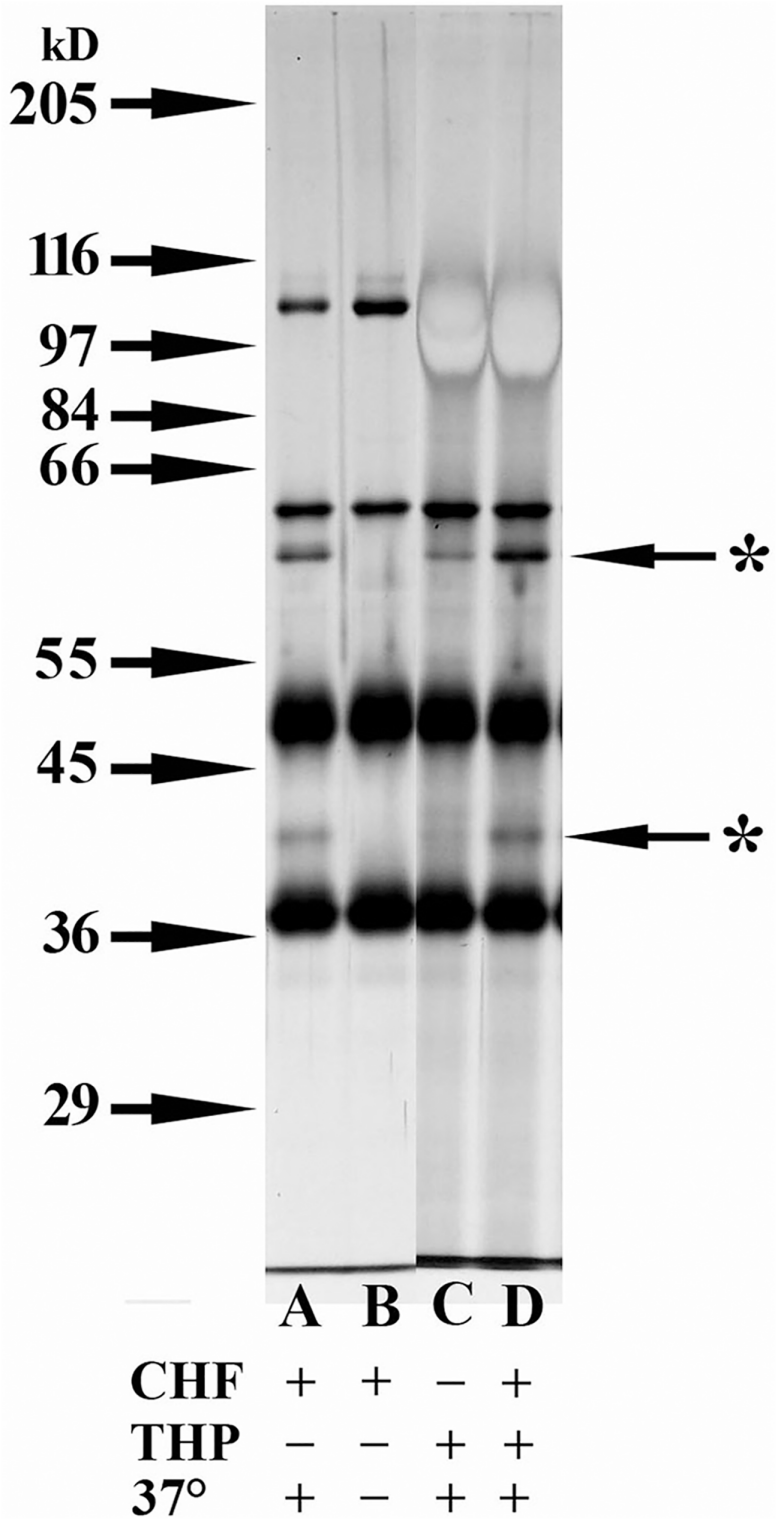
Cofactor activity present in samples with THP, but without CFH. Silver-stained 8% SDS-PAGE gel where all samples were reduced with 3% ß-mercaptoethanol. Samples analyzed in all lanes contained 125 ng C3b and 500 ng CFI. Lanes A, B, and D contained 0.5 ng CFH. Lanes C and D contained 5 μg THP. Lanes A, C, and D were incubated at 37°C for 30 min, while Lane B sample was kept at 4°C. Starred arrows indicate the degradation products of C3b.

In order to detect an effect of THP on the cofactor activity of CFH, the concentration of CFH was titrated to 1 ng CFH which resulted in some, but not complete, cleavage of C3b (see Fig A in S1 File). It was while performing C3b degradation experiments using this low concentration of CFH with varying amounts of THP where, very unexpectedly, in control samples where THP was added in the absence of CFH, degradation of C3b still occurred (Fig 3, Lane C), suggesting that the THP samples contained a cofactor for CFI-mediated C3b cleavage.

### Presence of C3b cofactor activity in multiple THP samples

**Fig 4:**
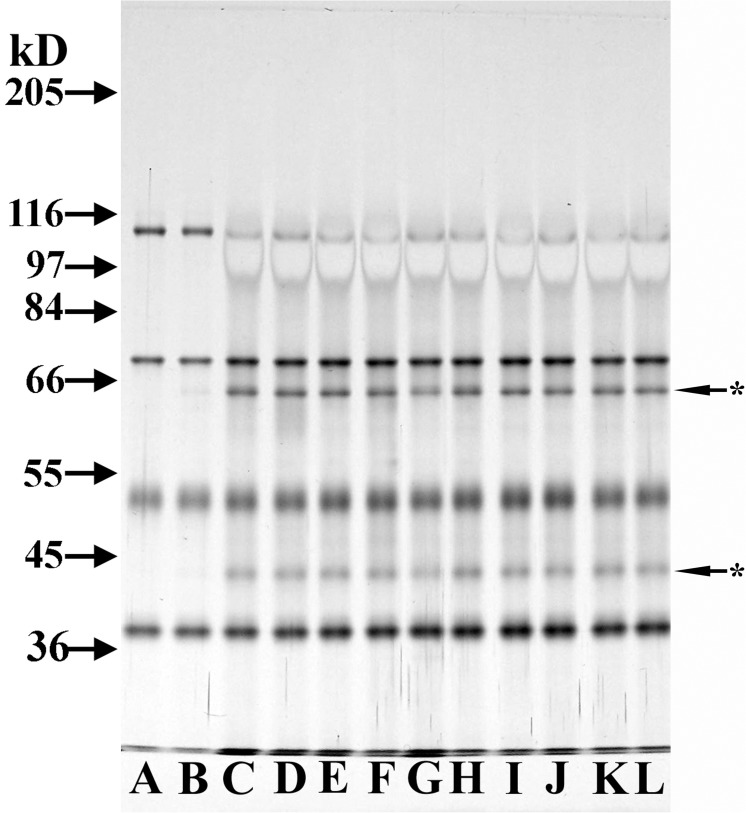
Comparison of cofactor activity in THP samples from 10 different individuals. 8% SDS-PAGE silver-stained gel of ß-mercaptoethanol-reduced samples. All lanes contained 125 ng C3b and 150 ng CFI. Lanes C → G samples contained 5 μg THP from 5 different females. Lanes H → L samples contained 5 μg THP from 5 different males. Samples in Lanes B → L were incubated at 37°C for 4 h. The sample in Lane A was not incubated at 37°C and was used to compare with the 37°C incubated sample in Lane B, both lanes containing only C3b and CFI. Starred arrows indicate the C3bα_62_ degradation product of C3b.

The universal nature of the cofactor activity of THP was assessed using THP samples from 5 females and 5 males. All 10 THP samples displayed significant cofactor activity (Fig 4). Since the intensity of the fragment bands would be related to the actual amount of C3b added to each tube, the intensity, as measured by Image J, of the C3bα_62_ and C3bα_41_ fragments was divided by the intensity of the C3b ß-chain (the chain not degraded in this reaction) in each lane to obtain a ratio. The ratio of C3bα_62_/C3b ß-chain was 0.565 ± 0.096 for the samples from females and 0.535 ± 0.087 for the male samples. The ratio involving the C3bα_41_ fragments was 0.397 ± 0.044 and 0.443 ± 0.050 for female and male samples respectively. No significant difference between male and female samples were detected for either band using the Students’ t-test (P>0.05). It was noted that the control sample containing only C3b and CFI still had a minor amount of C3b cleavage after a 4 h incubation at 37°C (Lane B); however, the ratios of the intensity of these bands to the C3b ß-chain were only 0.098 and 0.034 for the C3bα_62_ and C3bα_41_ fragments respectively.

### Evaluating THP samples for possible CFH contamination

One possibility for the cofactor activity in the THP samples was that THP might be contaminated with CFH. A sensitive, commercially-available ELISA was utilized to quantify CFH in all 10 THP samples and in C3b and several CFI samples. The standard curve (using CFH supplied by the ELISA manufacturer) was used to predict CFH concentrations in the protein samples (see Fig C in S1 File). No CFH was detected in the C3b or CFI samples tested; however, all 10 THP samples had very small, but detectable, amounts of CFH present in these, fairly concentrated, 1 mg/ml THP samples. In cofactor assays such those in Fig 4, only 5 μg of THP was loaded in each lane. When the amount of contaminating CFH in each 5 μg of THP was calculated, these values varied from 1.1 pg to 6.2 pg (see Fig C in S1 File). Despite this up to 6-fold difference in CFH in various THP samples, there was no correlation between the amount of contaminating CFH in the THP samples, as detected with the CFH ELISA, and the cofactor activity of THP, as measured by the ratios of C3bα_62_/C3b ß-chain for those THP samples in Fig 4 (r = -.0116, p = .975).

### Further characterization of the THP cofactor activity on C3b

**Fig 5:**
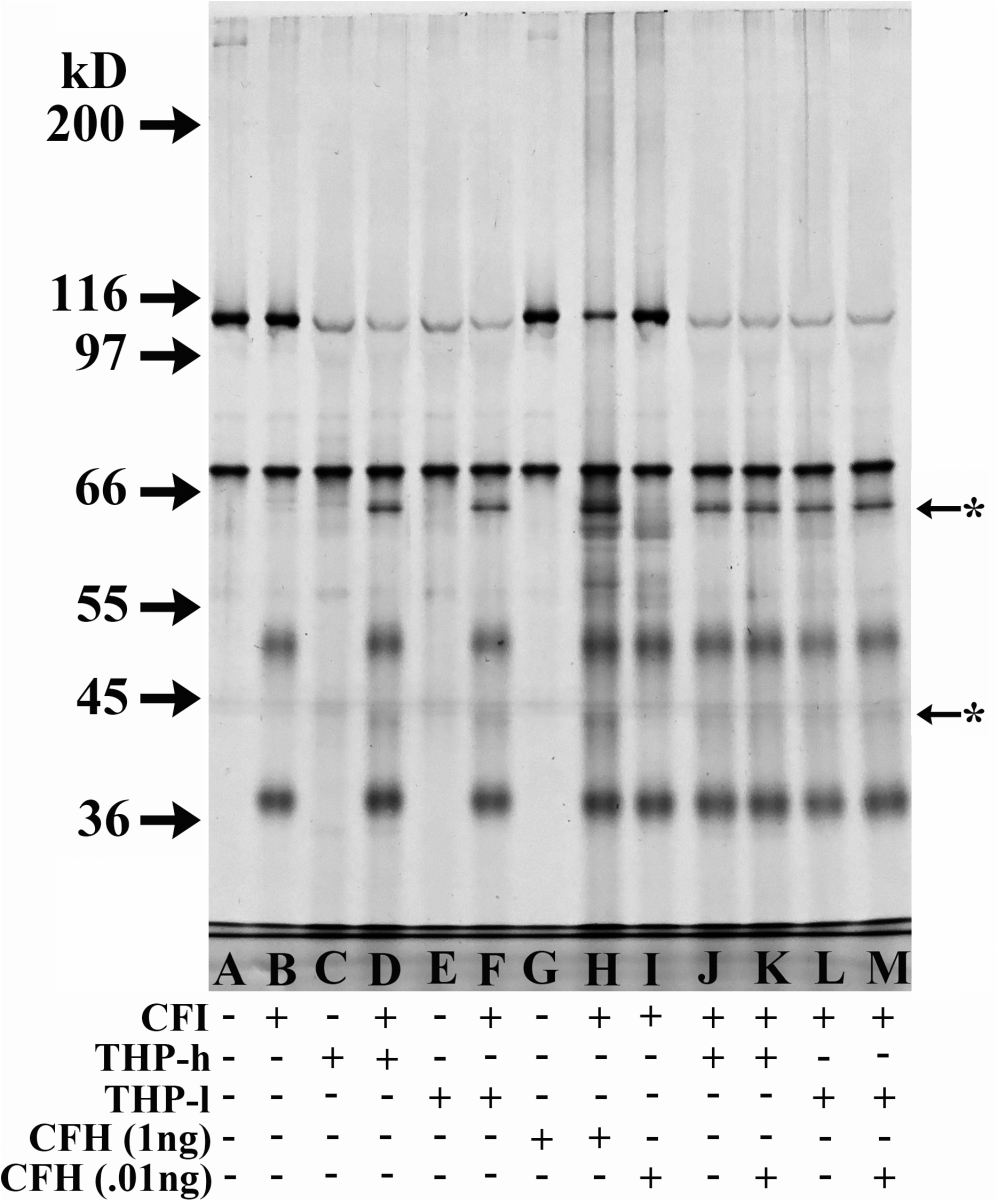
Comparison of cofactor activity in two THP samples with different amounts of contaminant CFH, and inability of small amounts of CFH to act as a cofactor. 8% SDS-PAGE, silver-stained gel of DTT-reduced samples. All cofactor assay samples were incubated at 37°C for 4 h. All lanes contained 125 ng C3b. Lanes B, D, F, and H→M contained 150 ng CFI. THP-h sample, by ELISA, had a predicted 5.3 pg CFH in 5 μg THP–and half of this amount was loaded in lanes C, D, J, and K. THP-l sample, by ELISA, had 1.1 pg CFH in 5 μg THP, and half of this amount was loaded into lanes E, F, L, and M. Lanes G and H were loaded with half the assay sample with 1 ng CFH and lanes I, K, and M had half of the assay sample with 0.01 ng CFH added.

Similar cofactor activity in various THP samples despite these samples having dissimilar CFH levels was confirmed when a “high” CFH THP sample (5.3 pg CFH/5 μg THP) was compared to a “low” CFH THP sample (1.1 pg CFH/ 5 μg THP). Image J analysis of the ratio of C3bα_62_/C3b ß-chain bands in both THP samples yielded the same ratio (0.36 versus 0.37) (Fig 5, Lanes D and J versus F and L). When 0.01 ng CFH, an amount equivalent to about twice the amount present in the “high CFH” 5 μg THP sample, was added to C3b and FI, no degradation of C3b was noted (Fig 5, Lane I). When this same amount of CFH (0.01 ng) was added to the two THP samples to determine if perhaps THP acted to potentiate CFH activity in this minute amount of CFH, no increased cofactor activity was noted (Fig 5 compare lanes J to K and L to M–and confirmed by Image J analysis of degradation bands).

**Fig 6:**
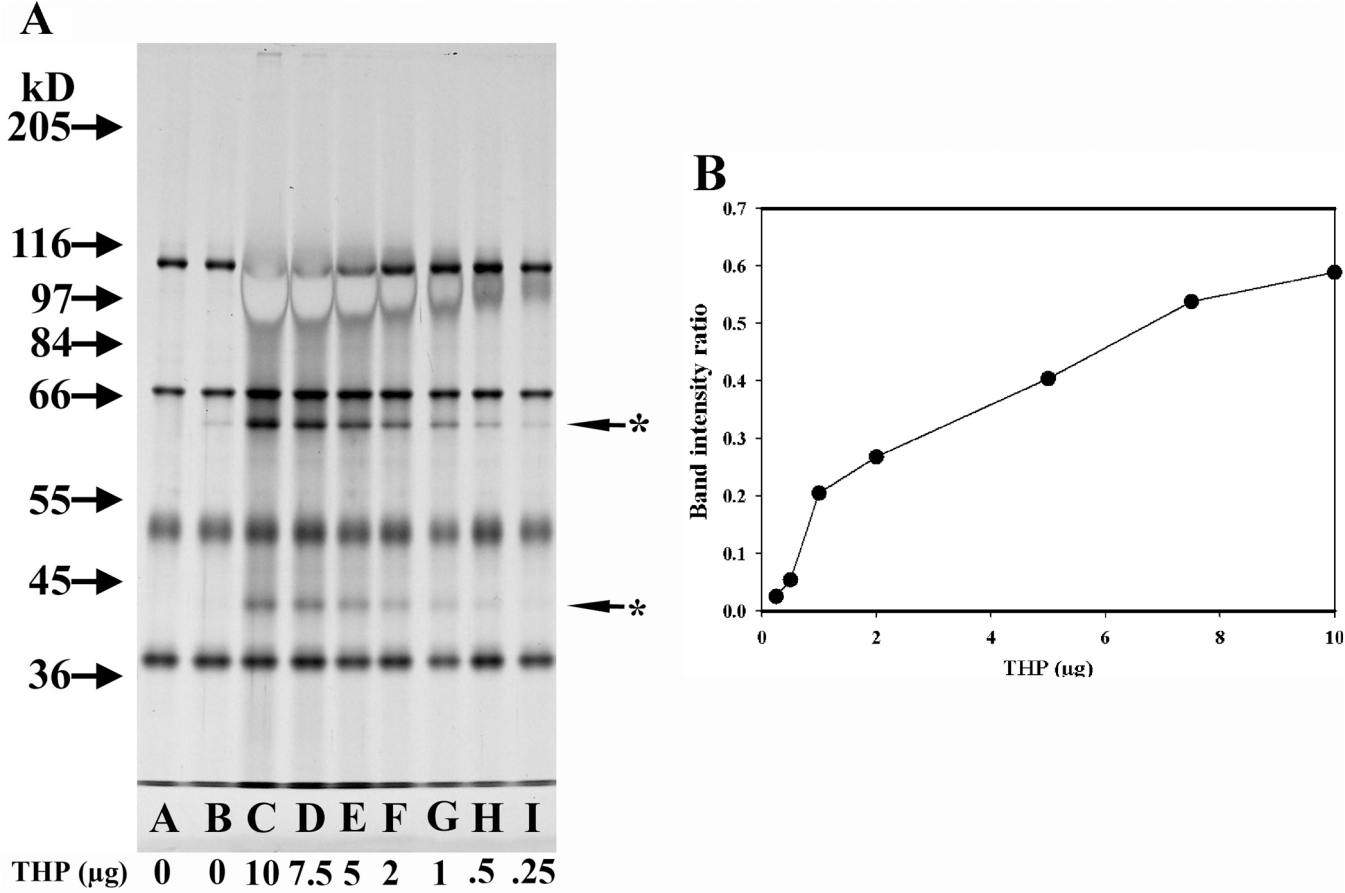
Dose effect of THP concentration on cofactor activity. (A) 8% SDS-PAGE silver-stained gel of ß-mercaptoethanol-reduced samples. All lanes were loaded with 125 ng C3b and 150 ng CFI. Samples in all lanes except that of Lane A were incubated at 37°C for 4 h. Lanes C → I contained samples with varying concentrations of a male THP sample (from 10 μg to 0.25 μg). Starred arrows indicate the degradation products of C3b. (B) Plot of THP quantity versus intensity of band ratio obtained after Image J analysis of the intensity of the C3bα_62_ fragment versus the C3b ß-chain in each lane minus the background cleavage present in Lane B.

If THP has intrinsic cofactor activity towards C3b, then increasing the concentration of THP should similarly increase the amount of C3b cleavage, which is indeed what occurred (Fig 6A). Quantification of this response was obtained by using ImageJ to determine the intensity of the C3bα_62_ fragment and the C3b ß-chain in each lane, calculating the ratio of these intensities and then subtracting from that the background cleavage ratio (i.e. the intensity of the C3bα_62_ fragment to the C3b ß-chain in the “C3b/CFI only” at 37°C for 4 h sample (Fig 6A, Lane B). Plotting the amount of THP in each sample by this corrected intensity of band ratio demonstrated a clear dose-dependent relationship between THP concentration and C3b cleavage (Fig 6B).

**Fig 7:**
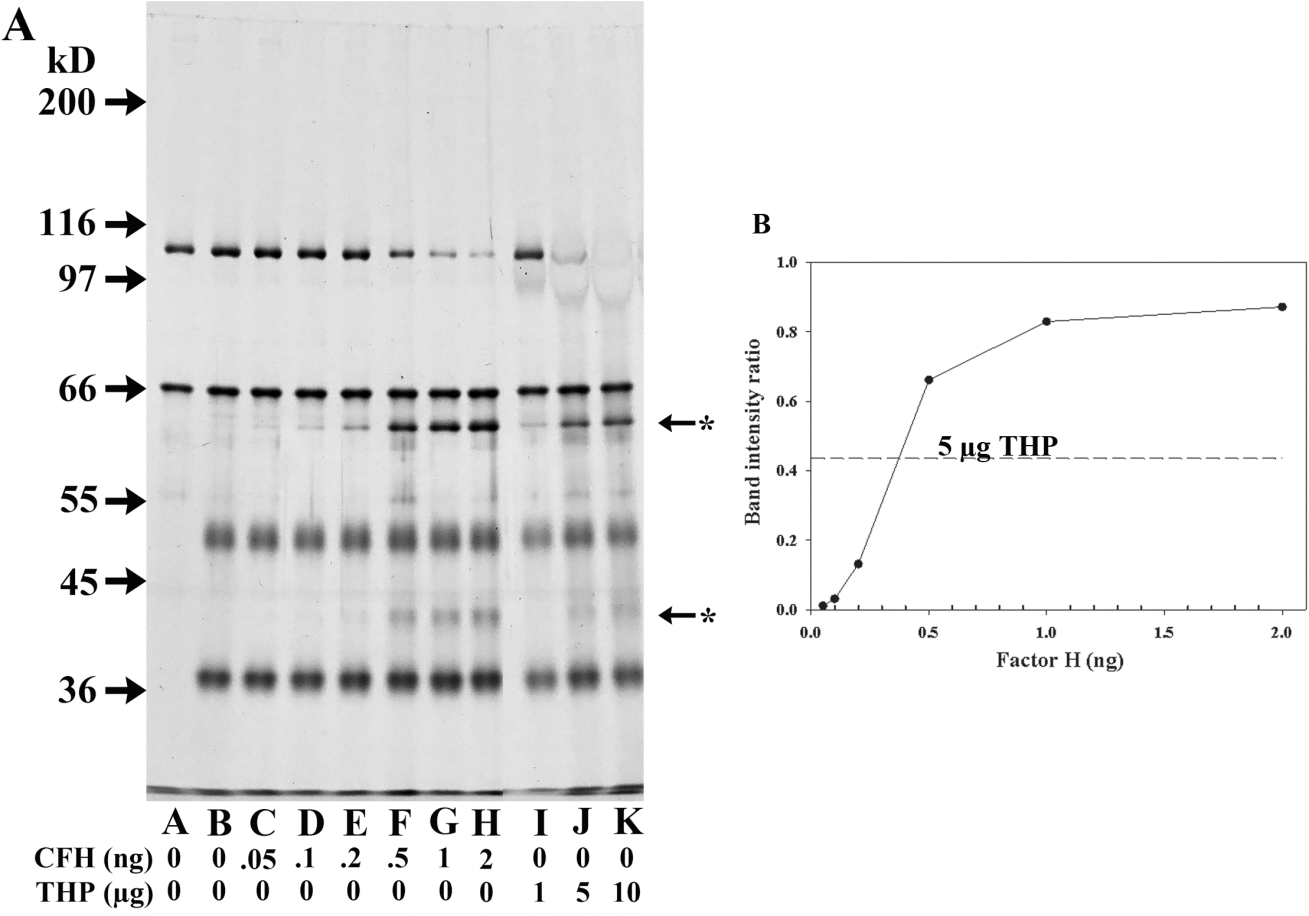
Dose effect of CFH concentration on cofactor activity and comparison with THP cofactor activity. (A) 8% SDS-PAGE silver-stained gel of DTT-reduced samples. All lanes had samples incubated at 37°C for 4 h and were loaded with 125 ng C3b and all lanes except Lane A contained 150 ng CFI. The chart below the lanes indicates the amount of CFH or THP present in the 10 μl samples, only half of which was loaded into the wells. Starred arrows indicate the degradation products of C3b. (B) Plot of CFH quantity versus intensity of band ratio obtained after Image J analysis of the intensity of the C3bα_62_ fragment versus the C3b ß-chain in each lane (C → H). Dashed horizontal line indicates the band intensity ratio present in the 5 μg THP sample (Lane J).

A similar dose-response cofactor assay was performed in 10 μl assay volumes with varying quantities of CFH (0.05 ng to 2.0 ng), and included 3 amounts of THP (1, 5, and 10 μg) in separate tubes to directly compare the cofactor activity of these two proteins. As in the preceding assay, quantification of the cofactor activity was obtained by using ImageJ to determine the intensity of the C3bα_62_ fragment and the C3b ß-chain in each lane and then calculating the ratio of these intensities. Since there was no detectable C3b cleavage in the “C3b/CFI only” sample (Fig 7A, Lane B), no background subtraction was needed. This assay effectively identified the useful range of CFH under these conditions, with the 0.05 ng and 0.1 ng CFH samples demonstrating only mild cofactor abilities, while the cofactor activity appeared to have peaked with the 2.0 ng sample (Fig 7A, Lanes C → H, and Fig 7B.) The samples with three different amounts of THP, showed differences in cofactor activity. As a rough comparison of cofactor efficiency between CFH and THP, the C3bα_62_ /C3b ß-chain band intensity ratio of the 5 μg THP was plotted as a dotted line of the CFH band intensity curve (Fig 7B). Extrapolation from this curve predicted that the amount of cofactor activity in 5 μg of THP is roughly equal to the activity of 0.37 ng CFH. From the CFH ELISA results, this particular THP sample was predicted to have about3.9 pg, that is 0.0039 ng, CFH which is 100-fold below the amount of CFH that would produce this effect.

**Fig 8:**
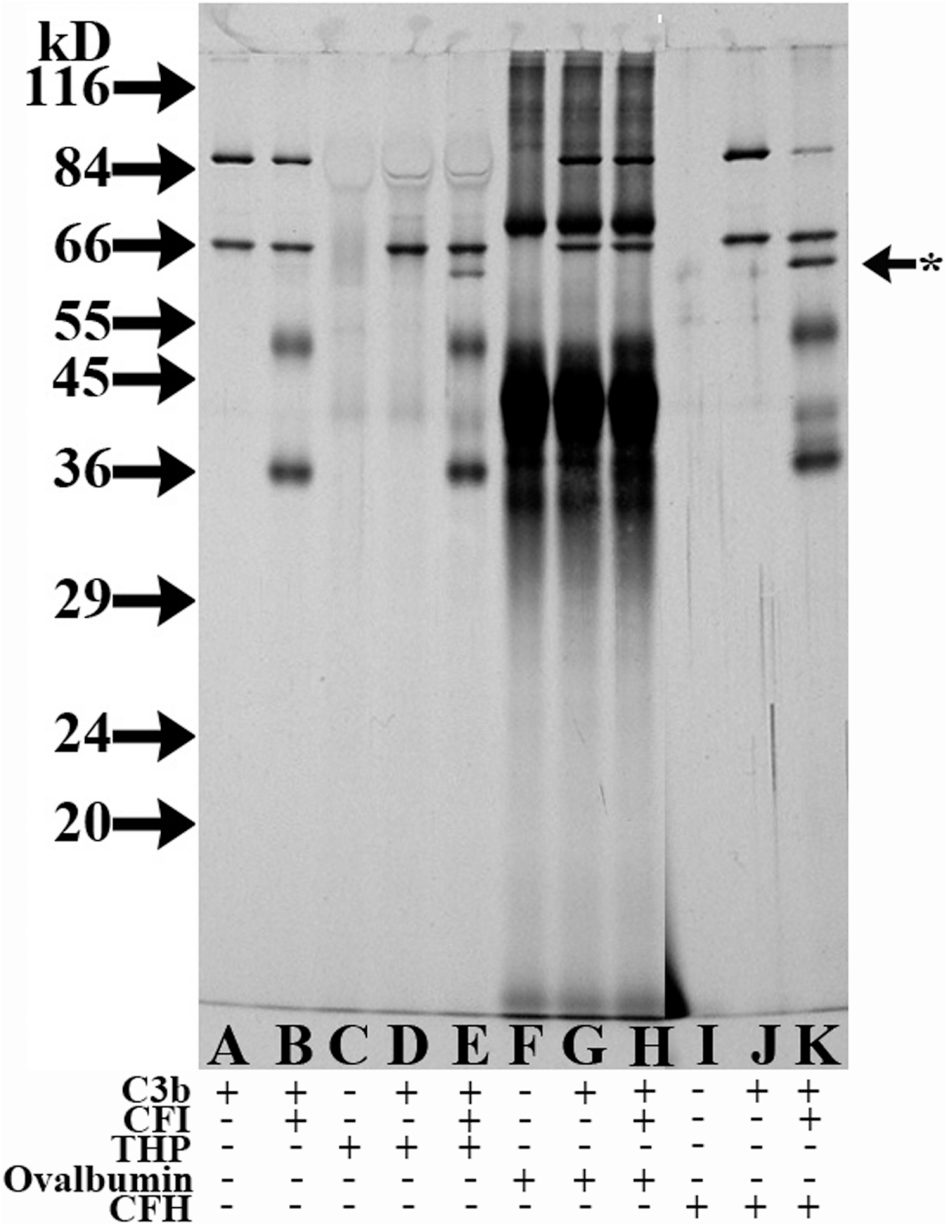
Evaluation for nonspecific protein-protein interactions causing cofactor activity. 12% SDS-PAGE silver-stained gel of DTT-reduced samples. Samples in all lanes were incubated at 37°C for 4 h. The chart below the gel indicates the proteins present in each lane, including C3b (125 ng), CFI (150 ng), THP (2.5 μg), ovalbumin (2.5 μg), and/or CFH (0.5 ng). Starred arrow indicates the C3b_α62_ degradation product of C3b.

While C3b cofactor activity was still detectable at low THP concentrations (even down to 0.25 μg THP, Fig 6), most of the assays in this study utilized 10 or 5 μg of THP in the 10 μl assay volume. To determine if the degradation of C3b simply was due to nonspecific protein to protein interactions from these relatively high protein concentrations, a cofactor assay compared 5 μg of ovalbumin to 5 μg THP in 10 μl cofactor assay volumes, with half of this volume analyzed by SDS-PAGE. While distinct C3b degradation bands were present in the sample with C3b, CFI, and THP (Fig 8, lane E) and in the C3b, CFI, CFH sample (Fig 8, lane K), no such bands were present in the ovalbumin sample when C3b and CFI were present (Fig 8, lane H).

### THP binding to C3b

**Fig 9:**
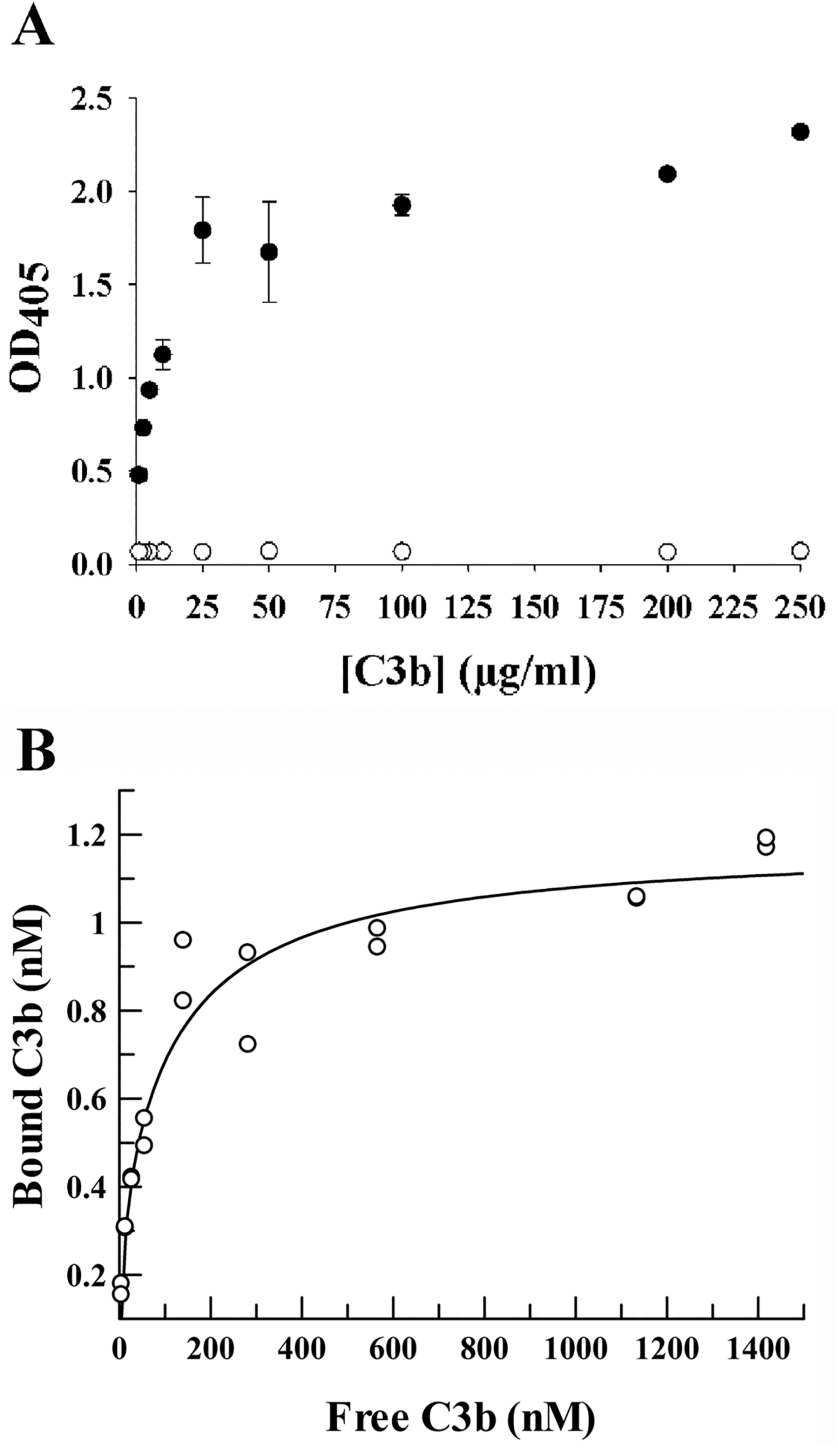
THP/C3b ELISA. (A) Plot of mean ± SD of absorbance of readings in duplicate wells indicating binding of various concentrations of C3b to THP-coated wells (solid circles) or uncoated wells (open circles). (B) Same data from the THP-coated wells plotted after fitting by non-linear least-squares analysis. K_D_ ~ 5 × 10^−8^ m. This estimate of K_D_ did not vary with the estimate of the amount of C3b bound to control wells. The K_D_ remained virtually unchanged whether it was assumed that 100% of added C3b bound to control wells or only 0.1% of C3b bound. Rather, the predicted number of binding sites decreased, in this case, by a factor of 1000.

In order for THP to function as a cofactor for CFI- mediated degradation of C3b, THP must bind C3b. The THP/C3b ELISA demonstrated that THP bound C3b in a dose dependent manner (Fig 9A). There was insignificant background binding of C3b to non-THP coated wells, indicating that blocking wells only with the 0.05% Tween in the buffer was sufficient to prevent nonspecific binding (Fig 9A). GraFit generated a binding curve (Fig 9B) with an estimated K_D_ of 5 × 10^−8^ M for the THP/C3b interaction.

### Effect of THP deglycosylation on cofactor activity

**Fig 10:**
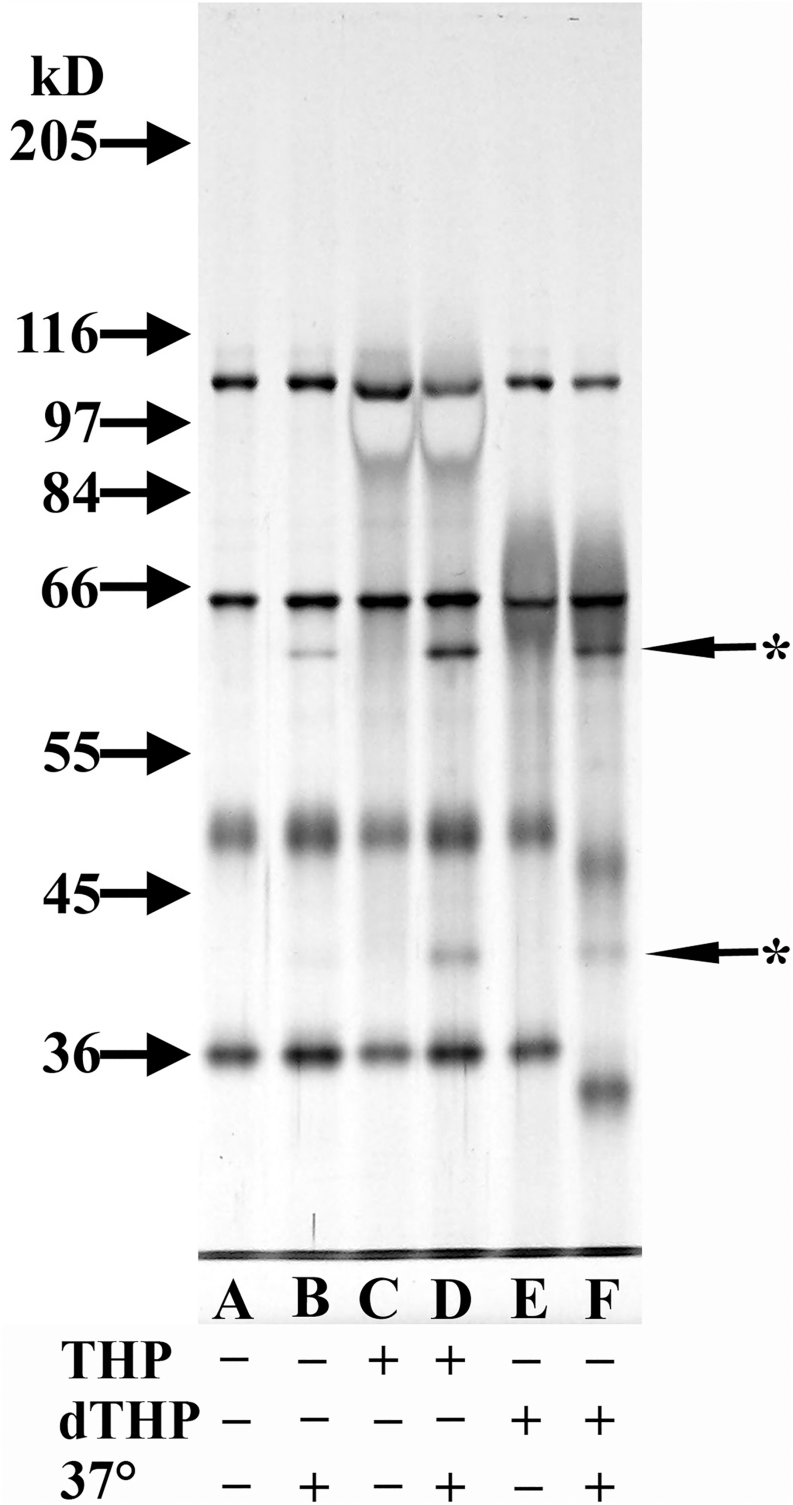
Analysis of cofactor activity in deglycosylated THP (dTHP). 8% SDS-PAGE silver-stained gel of ß-mercaptoethanol-reduced samples. All lanes were loaded with a mixture that contained 125 ng C3b and 150 ng CFI. Lanes C and D contained 3 μg THP while Lanes E and F contained what was estimated to be about 2 μg dTHP. Samples analyzed in Lanes B, D, and F were incubated at 37°C for 4 h while the other samples were not incubated at 37°C. Starred arrows indicate the degradation products of C3b.

Deglycosylated THP (dTHP), THP that had approximately 88% of its carbohydrate moieties removed enzymatically,[21] was evaluated for C3b cofactor activity. As seen in Fig 10, THP (Lane D) and dTHP (Lane F) yielded a higher concentration of C3b α-chain fragments than were present in the C3b/CFI sample (Lane B). While the THP sample had more intense fragment bands than did the dTHP sample, it was evident that more THP was present than dTHP, since higher THP concentrations stain more negatively.

## Discussion

We hypothesize that one function of THP is to act as an endogenous complement regulatory protein that can protect the kidney from unwanted complement activation. The present study was conducted to determine if THP interacted with CFH, a key complement regulatory molecule, since THP is negatively-charged and perhaps would interact with the polyanion binding sites of CFH.

In the ligand blots in the present study, THP recognized unreduced CFH that was at least partially denatured with the anionic detergent SDS in the sample buffer. However, complete linearization of CFH by reduction of its disulfide bonds abolished the CFH structure required for THP recognition.

The affinity of THP for CFH in the ELISA (K_D_ 1 × 10^−6^ M) is about 1000-fold weaker than THP’s binding to C1q which has a K_D_ of 10^−9^ M.[19,20] However, the affinity of CFH for THP is similar to the affinity of CFH for its normal ligand, C3b, which has been reported with a K_D_ of 10^−8^ M to a K_D_ of 10^−6^ M.[34,35] Another group recently published results similar to ours showing that THP/uromodulin bound to CFH using surface plasmon resonance (K_D =_ 4 × 10^−6^ M).[36]

In assessing the possible significance of an interaction between THP and CFH *in vivo*, it is important to realize that CFH is present in the kidney and urine, where THP normally is located. CFH expression occurs in normal renal cortical tubules[37] and recently, THP and CFH have been identified colocalizing in the renal tubules in IgA nephropathy patients.[36] While normal urinary CFH concentrations are low (0–14 U ml^-1^), increased CFH concentrations (ranging from 15 U ml^-1^ to 52,198 U ml^-1^) were detected in patients with a wide range of renal diseases.[38]

Besides colocalization of THP and CFH within the renal tubules and lower urinary tract, there may be a link between these two proteins at the transcriptional level. CFH knockout mice, which serve as a model for membranoproliferative glomerulonephritis, had significant changes in THP mRNA and protein concentrations. [39] Urinary THP concentrations were decreased 5.5-fold and THP mRNA levels were decreased 5.1-fold in these CFH knockout mice. Light microscopic examination of the kidneys revealed no significant tubulointerstitial disease in these animals, making it unlikely that the reduced THP expression was due to major tubular damage.

Having established that THP bound CFH, our study’s focus shifted to determining if this interaction altered the ability of CFH to downregulate complement by acting as a cofactor for CFI-mediated C3b proteolysis. As a control in these experiments, some samples contained THP, C3b, and CHI, but lacked CFH. These samples, free of added CFH, still possessed apparent cofactor ability since C3b degradation bands clearly were visible by SDS-PAGE. It is interesting to note that, in the recently published study on uromodulin and CFH, where the uromodulin was purified by a different technique than we used, their C3b degradation studies clearly showed C3b fragments in the control samples with THP, C3b, and CFI, but no CFH (Fig 6A, Lane 1 in reference #36), although the authors state that uromodulin had no effect on C3b inactivation without CFH.[36]

In the present study, we were intrigued by the possibility that THP could act as a cofactor in CHI-mediated C3b degradation. To test how universal this cofactor activity was, THP from 10 individuals was evaluated in the CFI-mediated C3b cofactor assay. All samples appeared very similar in their ability to act as cofactors.

Because CFH can be present in urine, and, as shown here, THP binds CFH, it was important to determine whether the apparent cofactor activity in these THP samples simply was due to contamination with CFH. The sensitive CFH ELISA did detect small amounts of CFH in each THP sample. However, there was no correlation between the amount of detected CFH in a THP sample and its cofactor ability. Furthermore, the amount of CFH in the THP samples was about 100-fold below the amount of CFH needed to produce the degree of cofactor activity present in THP.

Since all THP samples tested in the current study possessed a similar degree of cofactor activity, it is likely that this function resides in the protein portion of THP rather in the carbohydrate moieties which vary widely, even within an individual.[3,4] This hypothesis was supported when dTHP still possessed cofactor activity; however, our dTHP sample still had a small degree of glycosylation.[21] In other studies, the protein backbone of THP binds immunoglobulins,[18] while the carbohydrate moieties of THP impart other immunological properties.[9,10,21] Just as carbohydrates may not be required for THP’s cofactor activity, the N-glycans present on other complement regulatory proteins, such as C4-binding protein, decay accelerating factor (DAF, CD55) and CD59 are not required for their complement inhibitory activity.[40,41,42]

The dTHP samples had been generated by digesting THP with glycosidases. Analysis of the C3b cofactor studies with dTHP suggested that some glycosidase activity remained in these dTHP samples, since there was a molecular weight shift in the CFI protein chains after incubation at 37°C with the dTHP sample. CFI is heavily glycosylated with N-link oligosaccharide chains which are not required for its ability to cleave C3(NH_3_), a molecule functionally similar to C3b.[43] This correlated with the present study where removal of some carbohydrate from CFI did not significantly alter its proteolytic activity.

In some of our C3b cofactor assays, control samples containing only C3b and CFI that were incubated for several hours at 37°C did yield a low level of C3b proteolysis (Fig 4 Lane B and Fig 6 Lane B). Other studies also have shown a low level of proteolysis by CFI on C3b without known cofactors.[44,45] This low level of proteolysis by CFI did appear to be dependent on the specific CFI sample utilized, since these C3b degradation bands were not visualized in similar control wells in Figs 5,7 and 8 and Fig B in S1 File (lane B in all gels), where CFI from another supplier was used. It was this second group of CFI samples that was available for evaluation with the CFH ELISA and determined to be negative for this contaminant. Since the earlier CFI samples were not available for testing, it is possible that these CFI sample producing the low level of proteolysis was contaminated with a cofactor like CFH. However, the overall ability of THP to act as a cofactor in CFI-mediated C3b degradation was obvious in both sets of cofactor assays.

In order for THP to actually act as a cofactor for factor I-mediated C3b degradation, THP would need to bind to C3b, which was confirmed in our THP/C3b ELISA. The estimated binding affinity of THP for C3b compares favorably with the reported binding affinity of C3b to its native cofactor, CFH.[34]^,^[35]

When evaluating the possible significance of these *in vitro* cofactor assays, it is important to note that the concentrations of THP used were within the normal, physiologic range. The amount of THP excreted per day in the urine, and its concentration is highly variable. For example, 18–203 mg THP was quantified in 24-hour urine samples, with a mean of about 85 mg THP/24-hour and a mean absolute concentration of 61 ± 47 μg THP/ml of urine.[46] As seen in the Fig 6, 50 μg/ml THP (0.5 μg THP in a 10 μl cofactor assay sample) was able to act as a cofactor in C3b degradation.

Thus, while an important finding in the present study was the interaction between THP and CFH, a finding recently reported by others as well,[36] an even more significant finding was that THP acts as a cofactor in CFI-mediated C3b degradation. This finding supports our original hypothesis that THP can act as an endogenous complement regulator of the urinary tract. Since C3b lies at the convergence of all three arms of the complement pathways,[47] THP could down-regulate all three of these pathways by assisting with C3b cleavage.

Having an endogenous inhibitor of complement in the kidney would be advantageous in light of the detrimental role of complement in the pathogenesis of many forms of renal disease.[47,48,49] Although it is not an endogenous renal protein, vaccinia virus complement control protein (VCP) is a protein that, like THP, acts as a cofactor for CFI-mediated C3b degradation.[50] Treating rats with VCP in a model of IRI significantly improved kidney structure and function.[51] Demonstrating the importance of THP in a complement-mediated condition, THP knockout mice developed more severe renal damage when subjected to renal IRI than did wild type mice; wild type mice increased expression of THP in response to this injury and they redistributed THP towards the renal interstitium.[26,27] Hence, our finding that THP might down-regulate complement activation by assisting with cleavage of the key C3b molecule, suggests THP could play an important protective role in IRI and possibly other renal diseases involving complement activation. Future studies could evaluate the *in vivo* interactions between THP and the complement system using crosses of THP and C3 knockout mice with wildtype mice and evaluating their susceptibility to complement mediated renal disease models such as IRI. Additionally, future studies may be warranted that address how a complement regulatory function of THP may relate to the pathogenesis of autosomal dominant tubulointerstitial kidney disease, UMOD-related (ADTKD-UMOD), a chronic, progressive disease characterized by tubulointerstitial fibrosis in individuals with mutations in THP/uromodulin.[52]

## Supporting information

S1 File Additional supporting experiments and uncropped gels for figures in manuscript. (PDF)
