# Supplementary material for: Human Tamm-Horsfall protein, a renal specific protein, serves as a cofactor in complement 3b degradation
Source: PLoS One. 2017 Jul 24;12(7):e0181857. doi: 10.1371/journal.pone.0181857 (PMC5524369; doi:10.1371/journal.pone.0181857)
Supplement: S1 File — (PDF) [file pone.0181857.s001.pdf]

Human Tamm-Horsfall Protein, a Renal Specific Protein, Serves as a Cofactor in Complement 3b Degradation

Diana C.J. Rhodes, DVM, PhD*

Department of Anatomy, Pacific Northwest University of Health Sciences, Yakima Washington, 98901 United States

S1 File

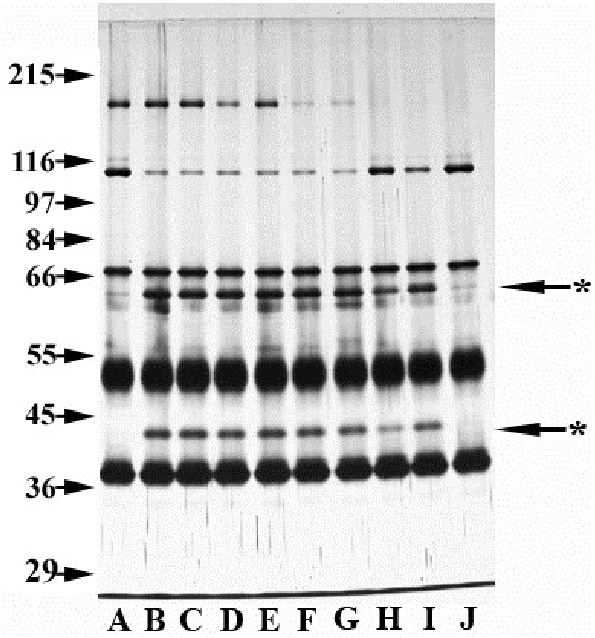

Figure A. CFH titration in cofactor assay. The concentration of CFH was titrated to determine the amount needed to cause some, but not complete, cleavage of C3b. Samples were reduced with 3% ß-mercaptoethanol in this 8% SDS-PAGE silver-stained gel. The original samples, each with a final volume of 10 µl for all lanes contained 250 ng C3b and 1 µg CFI while the original samples contained varying amounts of CFH: 100 ng CFH (Lanes A-C); 50 ng

CFH (Lanes D-E); 10 ng CFH (Lanes F-G), 1 ng CFH (Lanes H-I), or 0 ng CFH (Lane J). However, each lane was loaded with only half of these reaction mixtures, so the amount of CFH in the gel are half that listed. Lane A: control sample kept at 4°C. Lanes B, D, F, and H: incubated at 37°C for 10 min. Lanes C, E, G, and I: incubated at 37°C for 30 min. Lane J: control sample with only C3b and CFI (no CFH) incubated at 37°C for 30 min. Starred arrow indicates C3b degradation products, $C3b\alpha_{62}$ and $C3b\alpha_{41}$. The highest molecular weight protein on this gel, CFH (150 kD), is seen to be less prominent in lanes further to the right, which correlates with the lower amounts of CFH being present in these samples. In fact, in the 1 ng CFH samples (Lanes H and I) no CFH is visualized. However, even in these samples, there was some degradation of C3b into the $C3b\alpha_{62}$ and $C3b\alpha_{41}$ fragments during both 10 and 30 min. 37°C incubations. In contrast to Lanes B - G where more CFH was present, a significant amount of the 101 kD C3b α- chain remained uncleaved in these low-CFH samples (Lanes H-I). Thus, in most of the subsequent cofactor assays testing the influence of THP on this activity of CFH, 1 ng of CFH was utilized in the 10 μl reaction mixture, even though it was not visualized on the silver-stained gels.

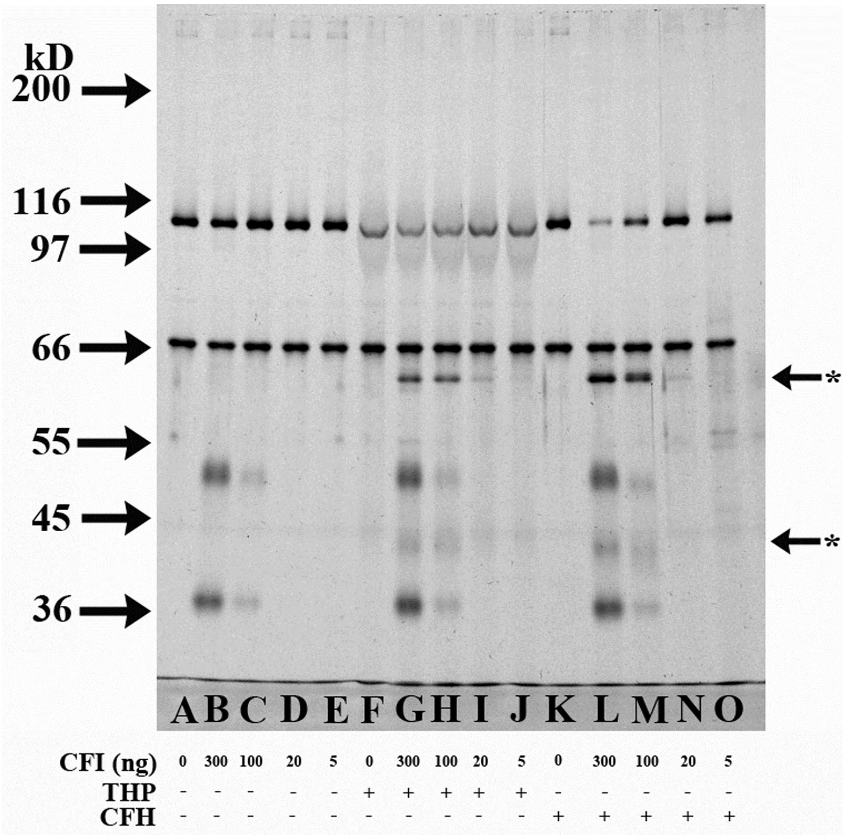

Figure B. CFI titration in cofactor assay. Similar to the CFH assay above (Figure A in S1 File), the effect of altering the concentration of CFI in this cofactor assay was assessed. All samples were incubated at 37 °C for 4 h before being reduced with 200 mM dithiothreitol for this 8% SDS-PAGE silver-stained gel. The original samples (total volume= 10 µl) for all wells contained 250 ng C3b. Some samples contained THP (5 µg) or CFH (1 ng). The amount of CFI varied from 300 ng to 5 ng (or 0 ng in control lanes). Each gel lane was loaded with half of these sample volumes. The C3b degradation bands, indicated by the starred arrows, were visualized in the samples with THP or CFH and CFI at 300ng, 100 ng, and 20 ng (Lanes G,H, I, L, M, N). No degradation of C3b was noted in the absence of THP or CFH (lanes (B → E) even with CFI present and no C3b cleavage was noted in when CFI was at 0 or 5 ng, even when THP (Lanes F and J) or CFH (Lanes K and O) was present.

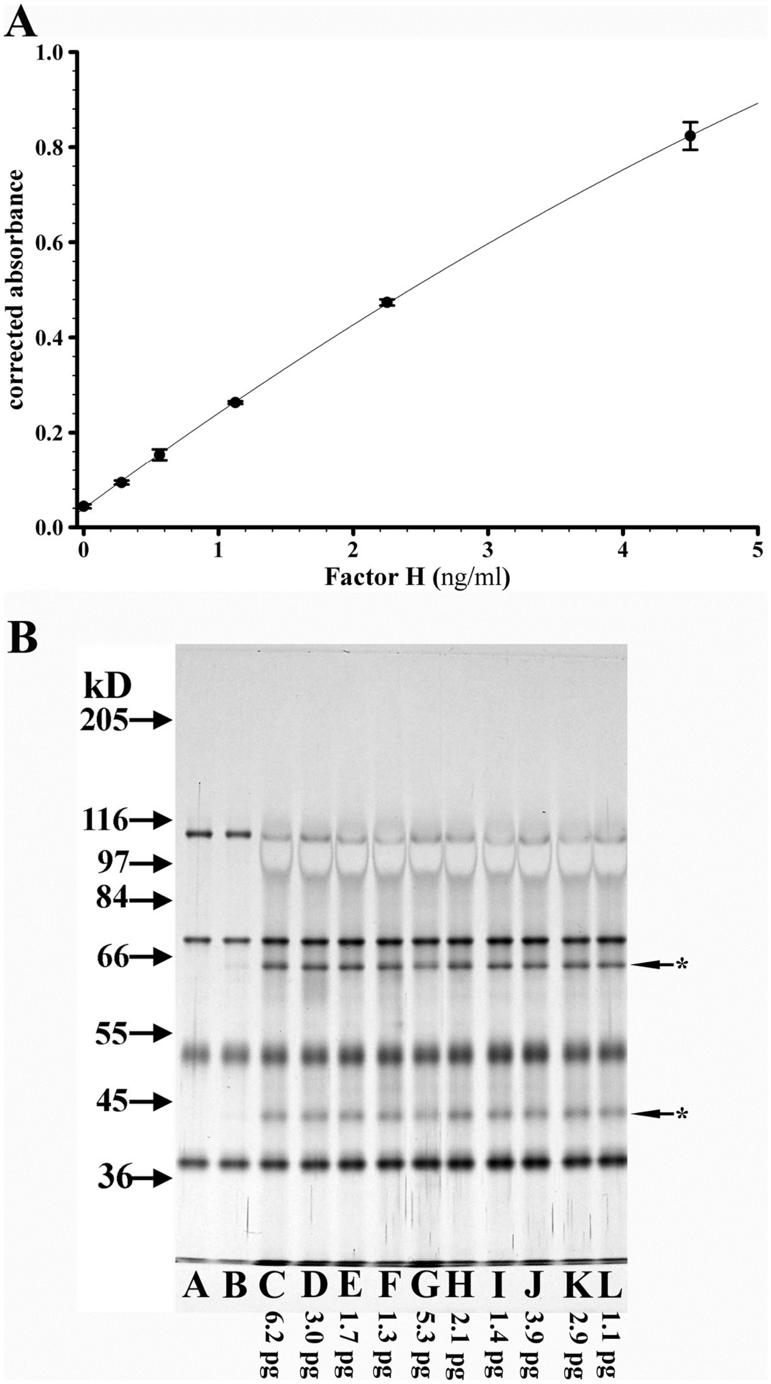

Figure C. Determination of CFH in THP samples. (A) Standard curve for CFH ELISA showing mean ± SD of duplicate samples. The line is that predicted by the 2nd-order polynomial regression (Sigma Plot) for standard samples < 5 ng/ml CFH. (B) Identical gel shown in Fig. 4

with the added label indicating the amount of contaminating CFH in each 5 µg sample THP used in individual cofactor assays.

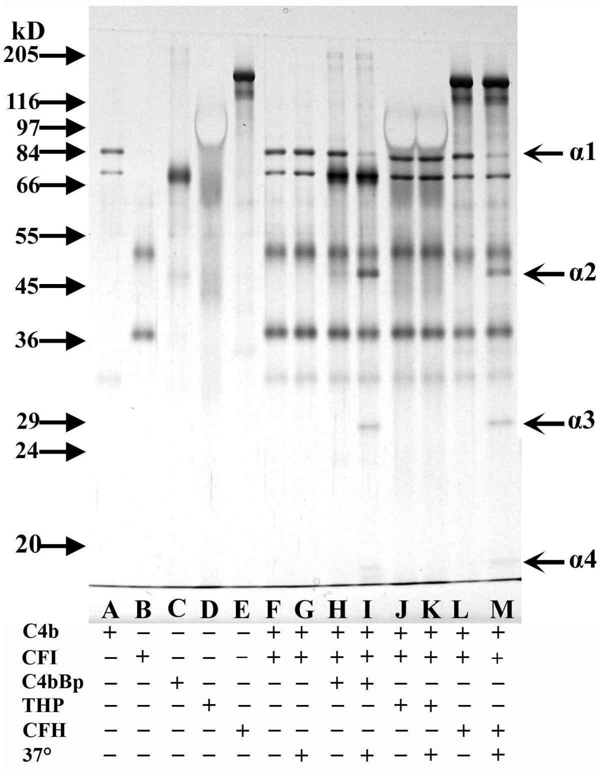

Figure D. C4b cofactor assay. Experiments were performed to determine if THP could act as a cofactor for CFI-mediated C4b cleavage. Each of the individual proteins in this assay, C4b, CFI, C4bBb, THP, and CFH, were analyzed in individual lanes (Lanes A → E) on a 10.5% SDS-PAGE, silver-stained gel. Lanes were loaded with half of the original sample mixture that contained combinations of 200 ng C4b, 200 ng CFI, 500 ng C4bBp, 10 µg THP, or 1 µg CFH. Samples were incubated at 37°C for either 0 h or 2 h before reduction with ß-mercaptoethanol and analysis on this gel. C4b cleavage products (indicated by the arrows labeled α2, α3, and α4),

and the subsequent decrease in the C4b α' band (labeled α1), were visualized only in the samples containing C4b/CFI + C4bBp (Lane I) and C4b/CFI + CFH (Lane M) that were incubated at 37°C for 2 h. C4b cleavage did not occur in samples that were not incubated at 37°C (Lanes H and L), nor were the C4b fragments noted in the "C4b/CFI only" (Lanes F and G) or the C4b/CFI + THP (Lanes J and K) samples incubated for 0 h or 2 h at 37°C. This assay was repeated three times with similar results. Thus, under these conditions, THP did not act as a cofactor for CFI-mediated C4b cleavage.

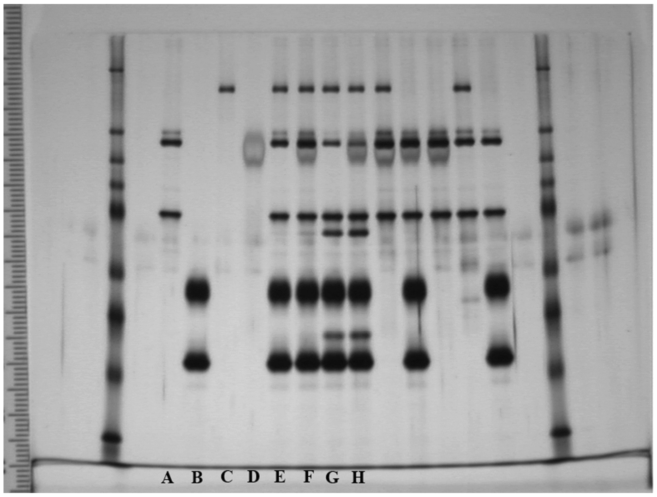

Figure E. Uncropped gel for Figure 2.

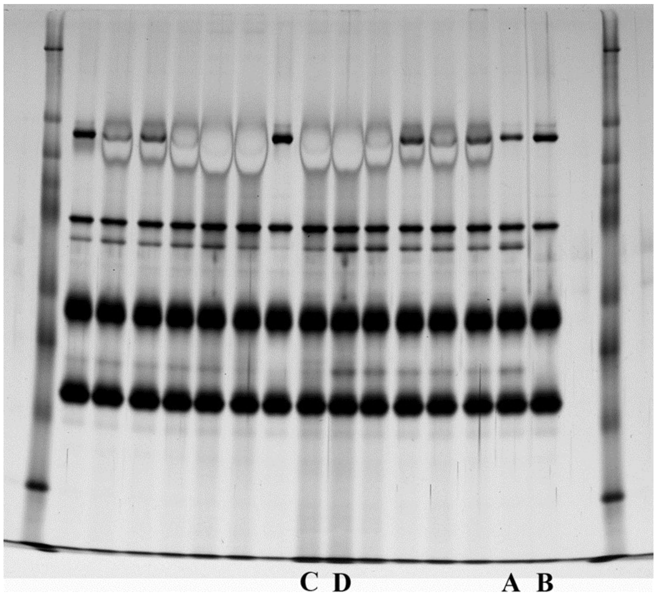

Figure F. Uncropped gel for Figure 3.

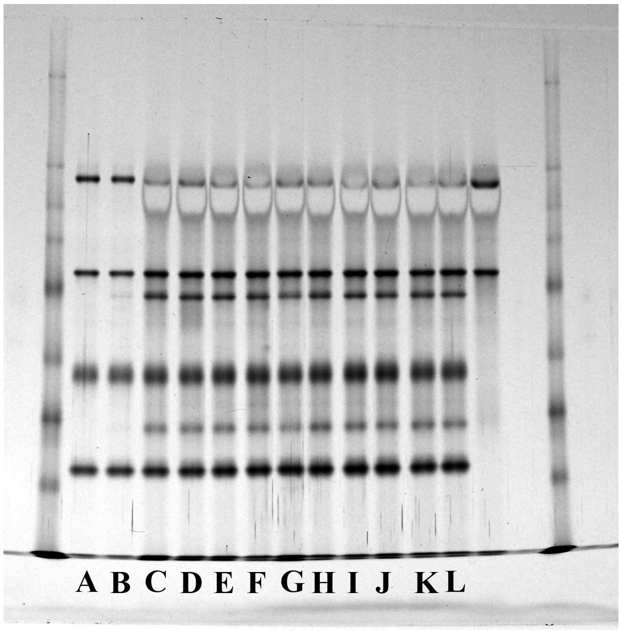

Figure G. Uncropped gel for Figure 4.

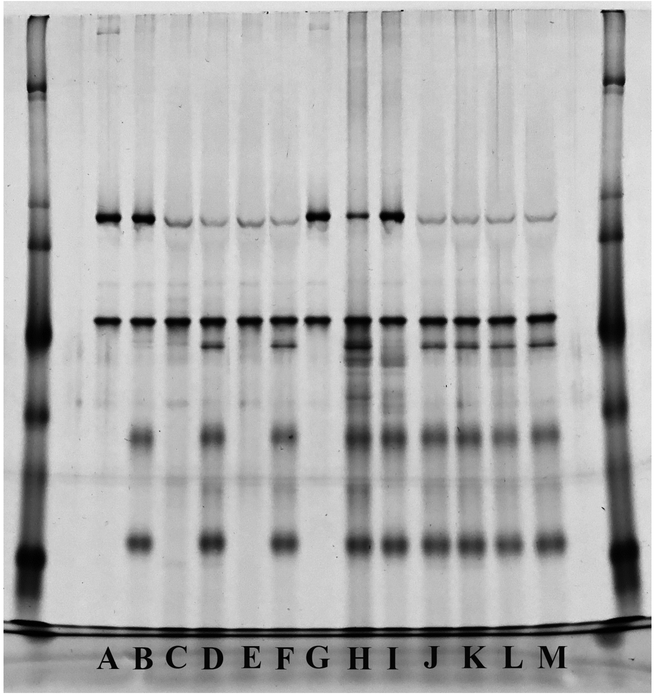

Figure H. Uncropped gel for Figure 5.

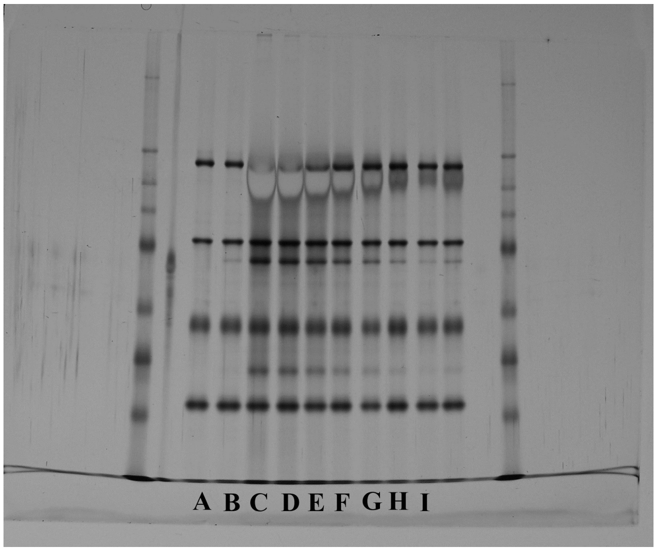

Figure I. Uncropped gel for Figure 6A.

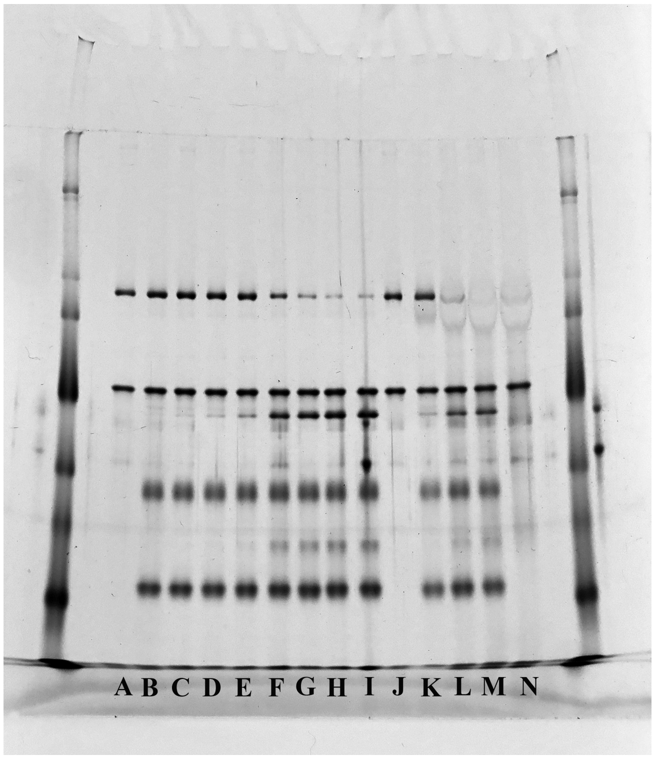

Figure J. Uncropped gel for Figure 7A.

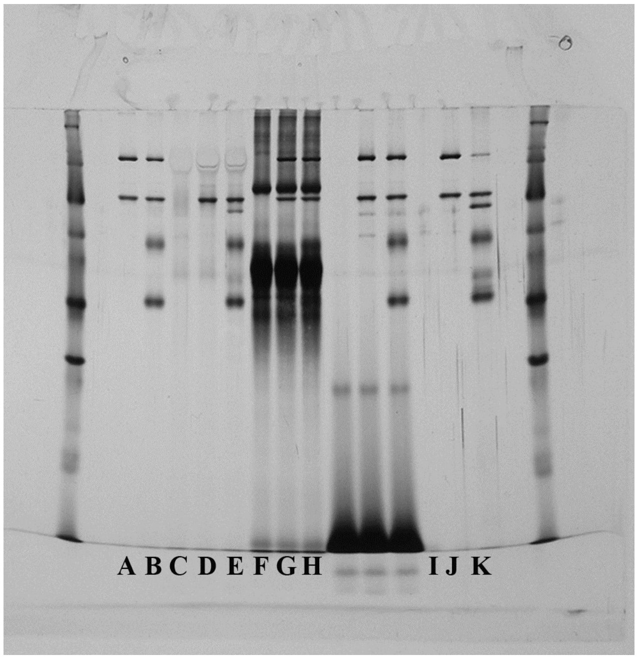

Figure K. Uncropped gel for Figure 8.

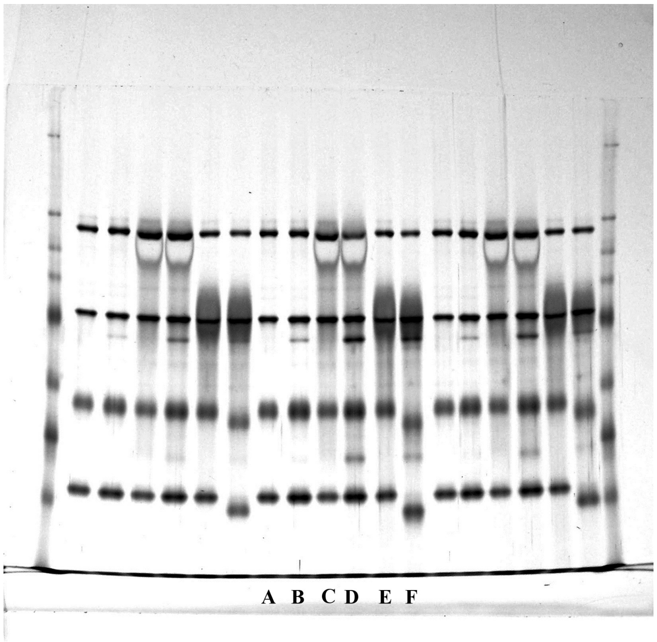

Figure L. Uncropped gel for Figure 10.
